# Cardiovascular and Mortality Outcomes in Lean Versus Non-lean Steatotic Liver Disease: A Systematic Review and Meta-Analysis

**DOI:** 10.7759/cureus.114731

**Published:** 2026-08-18

**Authors:** Husam Jawarneh, Zi Yong Goh, Mohammad F Alnoimat, Sanad Syam, Hadeel A Irshid, Lara Alqadi, Milad R Falahat, Noorhan M Al-Kadhimi, Mohammad Mustafa, Muath A Mustafa, Abdelkader O Rababah, Hala Alrababah, Adel Abdallah

**Affiliations:** 1 Family Medicine, King Abdullah University Hospital, Irbid, JOR; 2 College of Medicine, IMU University, Seremban, MYS; 3 College of Medicine, Jordan University of Science and Technology, Irbid, JOR; 4 College of Medicine, University of Jordan, Amman, JOR; 5 Internal Medicine, Jordan University Hospital, Amman, JOR; 6 Internal Medicine, University of Jordan, Amman, JOR; 7 Neurology, Mu’tah University, Mu’tah, JOR

**Keywords:** cardiovascular diseases, lean nafld, meta-analysis, metabolic dysfunction-associated steatotic liver disease, mortality, non-alcoholic fatty liver disease

## Abstract

Steatotic liver disease occurring at a normal body mass index is increasingly recognized, yet whether this phenotype carries a different prognosis from the non-lean phenotype remains contested. We aimed to compare cardiovascular events and mortality between lean and non-lean steatotic liver disease. PubMed, Scopus, and the Cochrane Library were searched from database inception to July 2026. Eligible studies were longitudinal cohorts reporting cardiovascular or mortality outcomes stratified by lean versus non-lean status. Because most contributing cohorts predate the 2023 nomenclature change, studies defined by nonalcoholic fatty liver disease (NAFLD) or metabolic dysfunction-associated fatty liver disease (MAFLD) criteria were retained without retrospective reclassification, and this was treated as a source of indirectness. Records were screened and data extracted in duplicate, and risk of bias was appraised using the Risk Of Bias In Non-randomized Studies of Interventions (ROBINS-I) with exposure-specific adaptation of its domains. Adjusted time-to-event estimates were preferred, transformed to the logarithmic scale, harmonized to a lean-versus-non-lean direction, and combined using random-effects inverse-variance meta-analysis with the DerSimonian-Laird estimator. Of 395 records identified, 265 were screened, 15 could not be retrieved, and 15 observational cohort studies were included, of which six contributed to at least one pooled analysis. Lean steatotic liver disease was associated with higher all-cause mortality (five studies; pooled hazard ratio (HR), 1.66; 95% CI, 1.48-1.86; P<0.001; I²=63.8%; τ²=0.009), a finding that persisted when restricted to the three studies providing a direct lean-versus-non-lean comparison (HR, 1.64; 95% CI, 1.42-1.90; P<0.001; I²=78.2%; τ²=0.011). Composite cardiovascular outcomes and major adverse cardiovascular events did not differ significantly (five studies; pooled HR/subdistribution hazard ratio (sHR), 0.92; 95% CI, 0.70-1.19; P=0.52; I²=90.7%; τ²=0.077); an HR-only sensitivity analysis excluding the single sHR was similarly null (four studies; HR, 0.96; 95% CI, 0.70-1.30; P=0.77; I²=88.1%; τ²=0.080). Heterogeneity was substantial, and these estimates are inconclusive rather than evidence of equivalence. Cardiovascular mortality was reported by two studies using non-identical effect measures and was not pooled: one study reported an HR of 1.40 (95% CI, 1.12-1.76), and the other reported an sHR of 0.94 (95% CI, 0.47-1.89). No study was rated as having low overall risk of bias; 10 were rated moderate and five serious. Lean steatotic liver disease appears to carry excess all-cause mortality, whereas evidence for cardiovascular event risk and cardiovascular mortality remains insufficient and heterogeneous. Applicability to contemporary MASLD should be interpreted cautiously because included cohorts used heterogeneous disease definitions and body mass index thresholds. The review was prospectively registered.

## Introduction and background

The nomenclature of fatty liver disease has changed twice within five years, and the sequence matters for how the present evidence base should be read. Nonalcoholic fatty liver disease (NAFLD), defined by exclusion of competing causes, gave way in 2020 to metabolic dysfunction-associated fatty liver disease (MAFLD), which introduced positive metabolic criteria [1]. In 2023, a multisociety Delphi process replaced both terms, establishing steatotic liver disease as an umbrella category and metabolic dysfunction-associated steatotic liver disease (MASLD) as the entity requiring hepatic steatosis together with at least one cardiometabolic risk factor [2]. The three definitions overlap substantially but not completely, and the cohorts that inform long-term prognosis were assembled under all three. Any synthesis of outcome data therefore combines studies whose case definitions differ in ways that cannot be fully reconciled retrospectively.

Steatotic liver disease is common, affecting roughly a quarter to a third of adults worldwide, and cardiovascular disease is a major cause of morbidity and mortality among affected individuals [3]. Most patients with MASLD die of cardiovascular disease or extrahepatic malignancy rather than of liver failure [4]. This has shifted clinical attention from hepatic endpoints toward cardiometabolic risk stratification, and it is the reason the prognosis of clinically atypical subgroups carries weight beyond hepatology.

From a public health perspective, even a minority lean phenotype is clinically important because a 5% to 20% lean fraction within a disorder affecting roughly one-quarter to one-third of adults represents a substantial number of patients worldwide [3,5]. The prognostic question is therefore not limited to nomenclature: if lean steatotic liver disease carries different mortality or cardiovascular risk, body mass index alone would be an inadequate basis for risk triage.

The lean phenotype is one such subgroup. Depending on the threshold applied, between 5% and 20% of patients with steatotic liver disease have a body mass index below 25 kg/m², or below 23 kg/m² where Asian-specific cutoffs are used [5]. These patients are not metabolically normal. A normal body mass index conceals visceral and ectopic adiposity, and lean patients with steatotic liver disease frequently demonstrate insulin resistance, adverse waist circumference, and reduced skeletal muscle mass [6].

Longitudinal comparisons of lean and non-lean cohorts have produced discordant results, and the discordance is not random. Studies differ in the body mass index threshold used to define leanness, in whether the comparator is a single non-lean group or a set of separate obesity classes, in whether the reference group is non-lean disease or no disease at all, in baseline diabetes prevalence and fibrosis stage, in follow-up duration, in the composition of cardiovascular endpoints, and in whether competing risks were modeled [7,8]. Several reports describe higher mortality among lean patients alongside variable cardiovascular event findings, a pattern difficult to interpret unless the outcomes are separated [7,9-12].

That pattern motivates the present review. Prior syntheses have frequently merged all-cause mortality, cardiovascular mortality, and cardiovascular events into a single composite, or have pooled odds ratios (ORs) alongside hazard ratios (HRs) to increase the number of contributing studies, both of which obscure the divergence [13,14]. We therefore analyzed these three outcomes separately, restricted quantitative pooling to compatible adjusted time-to-event estimates, and retained studies with incompatible effect measures or non-matching comparators for narrative synthesis rather than forcing them into a common model.

## Review

Methods

Protocol and Registration

The review protocol was registered prospectively on the Open Science Framework [15]. The review was prepared in accordance with the PRISMA 2020 statement [16].

Eligibility Criteria

Two levels of eligibility were applied and are distinguished throughout.

Eligibility for the systematic review: Eligible studies were prospective or retrospective longitudinal cohort studies involving adults aged 18 years or older with steatotic liver disease diagnosed by imaging, histology, or a validated biomarker score, stratified into lean and non-lean groups, and reporting cardiovascular events or mortality with an accompanying measure of precision. Cross-sectional studies, case-control studies, case series, reviews, and animal studies were excluded. Cohorts involving untreated secondary causes of hepatic steatosis, including significant alcohol consumption, viral hepatitis, and long-term use of steatogenic medications, were also excluded.

Eligibility for quantitative pooling: The target comparison for quantitative synthesis was lean versus non-lean steatotic liver disease. A compatible adjusted time-to-event estimate contrasting lean with non-lean participants was additionally required. When a study used obesity, overweight, or discrete body mass index strata rather than a single all-non-lean comparator, the estimate was treated as an indirect approximation of the target comparison and was included only if the direction could be harmonized without double-counting a shared reference group. The absence of a compatible adjusted time-to-event estimate did not exclude an otherwise eligible study from the review; such studies were retained for narrative synthesis. Studies reporting only ORs, crude incidence rates, event percentages, or estimates against a non-disease reference group were therefore included in the review but not pooled.

Cohorts defined by NAFLD or MAFLD criteria were retained without retrospective reclassification to MASLD, because the primary reports do not supply the participant-level cardiometabolic variables required to reapply the 2023 criteria. The included evidence is consequently a mixture of MASLD, MAFLD, and NAFLD cohorts, and the pooled estimates should be understood as applying to metabolically defined steatotic liver disease broadly rather than to MASLD as strictly defined.

Information Sources and Search Strategy

PubMed, Scopus, and the Cochrane Library were searched from database inception to July 2026. No date-of-publication restriction was applied. These databases were selected to capture biomedical indexing, multidisciplinary citation coverage, and controlled evidence-synthesis sources relevant to the review question. No other database was searched, and no register, website, or citation-searching source contributed records. Controlled vocabulary and free-text terms covering steatotic liver disease nomenclature, the lean phenotype, and cardiovascular and mortality endpoints were combined using Boolean operators and adapted to the syntax of each database. The search returned 129 records from PubMed, 253 from Scopus, and 13 from the Cochrane Library, giving 395 records in total, with 265 remaining after removal of 130 duplicates. Complete search strings are provided in the Appendices. The absence of supplementary citation searching and the inability to retrieve 15 reports are recognized as potential sources of missed eligible evidence and are therefore considered in the Limitations.

Study Selection

Records were de-duplicated and screened in two stages, first by title and abstract and subsequently by full text, independently by two reviewers using Rayyan (Rayyan Systems Inc., Cambridge, MA, USA). Disagreements were resolved by consensus or, where necessary, by adjudication from a third reviewer. Reports for which no full-text article could be obtained were recorded as not retrieved and were not assessed against the eligibility criteria. These unavailable reports were retained in the screening log rather than being counted as full-text exclusions.

Data Extraction

Two reviewers independently extracted study, population, exposure, outcome, and effect-estimate data into a piloted extraction form. Discrepancies were reconciled by consensus or third-reviewer adjudication.

Outcomes

Three outcomes were specified and analyzed separately and were not merged into a single composite outcome: all-cause mortality; composite cardiovascular outcomes and major adverse cardiovascular events (MACE); and cardiovascular mortality.

Risk-of-Bias Assessment

Risk of bias was assessed independently in duplicate using the Risk Of Bias In Non-randomized Studies of Interventions (ROBINS-I) tool [17]. Because this review addresses a prognostic exposure rather than an intervention, the domains were adapted to exposure assessment and relabeled accordingly: bias due to confounding (D1); bias in selection of participants (D2); bias in classification of the exposure (D3), referring to the classification of participants as lean versus non-lean steatotic liver disease; bias due to deviations after exposure classification or differential post-exposure management (D4), referring to differences in management or care occurring after lean versus non-lean classification; bias due to missing data (D5); bias in measurement of outcomes (D6); and bias in selection of the reported result (D7). Prespecified important confounders were age, sex, smoking, alcohol consumption, diabetes, hypertension, dyslipidemia, and fibrosis severity. Each study received an overall judgment of low, moderate, serious, or critical risk of bias, taken as the least favourable domain judgment. These exposure-specific interpretations of D3 and D4 were used only to adapt ROBINS-I to an exposure study and did not alter the risk-of-bias judgments.

Risk Of Bias In Non-randomized Studies-of Exposures (ROBINS-E) was not used because ROBINS-I was prespecified in the registered protocol; changing tools post hoc would have constituted an undocumented protocol deviation. The exposure-specific adaptation described above is reported as a documented modification. ROBINS-E would be the preferable instrument for future reviews of this question.

Statistical Analysis

Adjusted time-to-event estimates were preferred. Estimates were transformed to the logarithmic scale and standard errors (SEs) derived from the reported 95% confidence intervals (CI) as $SE = \frac{\ln(\text{upper CI}) - \ln(\text{lower CI})}{3.92}$. All estimates were harmonized to a lean-versus-non-lean direction, such that values greater than 1 indicate higher risk in the lean group; estimates reported in the reverse direction were inverted together with their confidence limits. Random-effects inverse-variance meta-analysis was performed using the DerSimonian-Laird estimator of between-study variance, as prespecified in the analytic plan. This estimator was retained as the primary model to preserve consistency with the registered analytic approach and because the quantitative syntheses were exploratory and based on few studies. Given the small number of contributing studies, all pooled intervals were interpreted cautiously. As a post hoc robustness check, Hartung-Knapp-Sidik-Jonkman CIs were also calculated for pooled outcomes and interpreted qualitatively. Heterogeneity was quantified using the Cochran Q statistic, I², and τ².

ORs and unadjusted incidence-rate ratios were not pooled with adjusted hazard estimates. Studies reporting only separate body mass index-class estimates against a shared lean reference group were not mathematically recombined, since doing so would double-count the reference group. HRs and subdistribution hazard ratios (sHRs) estimate different parameters in the presence of competing risks; for cardiovascular mortality, where the only two available estimates were of these two types, no pooled estimate was calculated, and the two results are reported separately. For composite cardiovascular outcomes, where four Cox HRs were available alongside one sHR, the mixed HR/sHR analysis is reported because it preserves the most complete set of adjusted time-to-event estimates relevant to the target endpoint; however, the HR-only sensitivity analysis is given equal interpretive weight, and the mixed estimate is not interpreted as definitive.

Potential overlap of study populations was assessed before quantitative synthesis by comparing data sources, institutions, recruitment periods, and eligibility criteria rather than authorship alone. Two forms of potential overlap were identified and are reported in Results. Where overlap was present, at most one report contributed to any single pooled analysis.

Sensitivity Analyses

Two prespecified sensitivity analyses were performed. For all-cause mortality, the model was restricted to studies providing a direct lean-versus-non-lean comparison, excluding those whose comparator was defined by body mass index class. For composite cardiovascular outcomes, the model was restricted to conventional Cox HRs, excluding the single sHR. A post hoc Hartung-Knapp-Sidik-Jonkman sensitivity analysis was also performed for pooled outcomes to assess the influence of small-study random-effects inference. Subgroup analyses by disease definition, body mass index threshold, or region were not undertaken because each pooled outcome contained only three to five contributing studies and because several studies differed simultaneously across multiple classification features. No leave-one-out analysis or meta-regression was undertaken.

Small-Study Effects

Formal testing for small-study effects and publication bias was not undertaken, as no outcome was informed by the minimum of 10 contributing studies at which such tests carry adequate power. No funnel plot, Egger test, Begg test, or trim-and-fill analysis was performed.

Software

Statistical analyses were performed in Python (version 3.12; Python Software Foundation, Fredericksburg, VA, USA) using the statsmodels package (version 0.14.4).

Results

Study Selection

**Figure 1:**
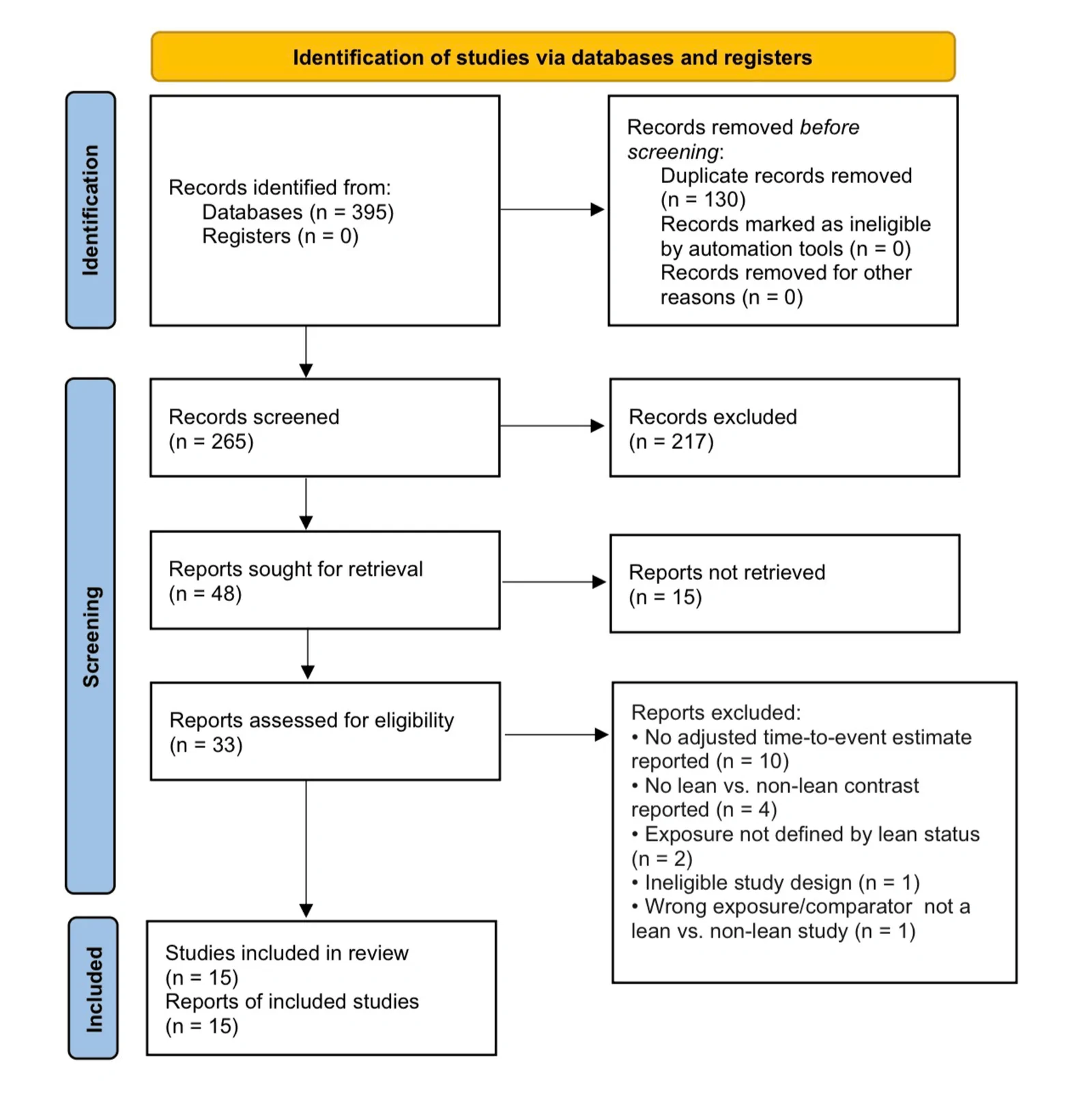
PRISMA 2020 flow diagram of study selection.

The flow of records through identification, screening, eligibility, and inclusion is shown in Figure 1. The database searches identified 395 records, including 129 from PubMed, 253 from Scopus, and 13 from the Cochrane Library. After removal of 130 duplicates, 265 records were screened by title and abstract, of which 217 were excluded. Forty-eight reports were sought for retrieval; 15 could not be obtained despite full-text retrieval attempts, leaving 33 reports assessed for eligibility. Eighteen reports were excluded at full-text assessment. Fifteen observational cohort studies met the eligibility criteria and were included in the systematic review. Six studies contributed to at least one quantitative synthesis, while nine studies were retained for narrative synthesis only.

Two record-level corrections were made during study selection. One record previously entered twice under two author labels (Arvind, 2022 and Ishido, 2023) was identified as a single study and consolidated under the verified citation Ishido et al. [18]. A second record previously listed under the label Aboona (2024) was found not to meet the eligibility criteria and was excluded at full-text assessment; it is counted among the 18 full-text exclusions in Figure 1 and Table 1 under the reason “wrong exposure/comparator.”

**Table 1:** Reports excluded at full-text assessment, with one primary reason per report. Reference numbers are given for each excluded report. IBD: inflammatory bowel disease

Report (first author, year)	Primary reason for exclusion
Weinberg (2021) [19]	No adjusted time-to-event estimate: reports prevalence and odds ratios for cirrhosis, cardiovascular disease, and metabolic abnormalities
Dou (2025) [20]	No adjusted time-to-event estimate: cardiovascular outcomes reported as percentages only
Almomani (2023) [21]	No adjusted time-to-event estimate: acute coronary syndrome reported as a percentage
Semmler (2021) [22]	No adjusted time-to-event estimate: cardiovascular mortality reported only as a proportion of deaths
Biswas (2023) [23]	No adjusted time-to-event estimate: multivariate analysis reports odds ratios rather than hazard ratios
Ye (2025) [24]	No adjusted time-to-event estimate: cause of death after follow-up not described
Iritani (2020) [25]	No adjusted time-to-event estimate: reports incidence rates and Kaplan-Meier survival only
Fracanzani (2017) [26]	No adjusted time-to-event estimate: cardiovascular results derive from baseline data rather than incident events
Feldman (2021) [27]	No adjusted time-to-event estimate: cardiovascular deaths reported as counts only
Zou (2020) [28]	No adjusted time-to-event estimate: does not report the required effect estimate for incident cardiovascular outcomes
Charatcharoenwitthaya (2024) [29]	No lean-versus-non-lean contrast: mortality analysis focused on all-cause mortality and alcohol interaction
Behari (2024) [30]	No lean-versus-non-lean contrast: all-cause mortality reported without cardiovascular data
Alarabi (2026) [31]	No lean-versus-non-lean contrast: participants grouped by DNAm-GDF15 level
Zhang (2024) [32]	No lean-versus-non-lean contrast: participants grouped according to type 2 diabetes mellitus
Aneni (2020) [33]	Exposure not defined by lean status: outcome is cardiometabolic disease rather than cardiovascular events or mortality
Zhang (2025, IBD cohort) [34]	Exposure not defined by lean status: cohort of patients with inflammatory bowel disease subsequently subclassified
Zhu (2025) [35]	Ineligible study design: cross-sectional
Aboona (2024) [36]	Wrong exposure/comparator: ethnicity-based rather than lean-versus-non-lean analysis

Study Characteristics

The 15 included cohorts were published between 2017 and 2026 and were conducted in the United States (six studies), China (two), South Korea (two), Japan (two), Italy (one), the United Kingdom (one), and one multinational database study. Retrospective cohorts predominated, with prospective designs contributing the largest population-based datasets. Follow-up ranged from three years to a mean of 22.4 years.

Definitions of leanness were not uniform. Studies in Asian populations generally applied a threshold of body mass index below 23 kg/m², whereas Western cohorts used below 25 kg/m²; several multicenter studies from the United States applied an ethnicity-specific rule of below 25 kg/m², or below 23 kg/m² in Asian participants. Diagnostic terminology likewise varied: cohorts applied contemporary MASLD criteria, MAFLD definitions, and historical NAFLD definitions. Hepatic steatosis was ascertained predominantly by ultrasonography, with diagnostic coding from electronic health records used in the multi-institutional database studies. Adjustment sets typically included age, sex, diabetes, hypertension, and dyslipidemia, whereas smoking status and fibrosis stage were adjusted for inconsistently.

No aggregate participant total is reported, because two included cohorts draw on partially overlapping institutional populations, as described below. Summing cohort sizes would double-count participants. The principal characteristics of the included studies are summarized in Table 2.

**Table 2:** Characteristics of included studies, listed chronologically. ASCVD: atherosclerotic cardiovascular disease; BMI: body mass index; CHD: coronary heart disease; CVD: cardiovascular disease; MACE: major adverse cardiovascular events; VA: Veterans Affairs; WC: waist circumference; NHANES: National Health and Nutrition Examination Survey; NHIS: National Health Insurance Service; MASLD: metabolic dysfunction-associated steatotic liver disease; MAFLD: metabolic dysfunction-associated fatty liver disease; NAFLD: nonalcoholic fatty liver disease

Study (year)	Country	Design	N	Lean BMI threshold	Terminology	Follow-up	Outcomes reported
Hashimoto (2017) [37]	Japan	Prospective (post hoc)	1,647	<23 kg/m²	NAFLD	5 years	Incident CVD
Golabi (2020) [38]	USA	Longitudinal (NHANES III, 1988-1994)	9,341	BMI × WC phenotypes	NAFLD	Mean 22.4 years	All-cause, CV, cancer mortality
Ahmed (2022) [9]	USA	Population-based retrospective (Olmsted County)	4,834	Normal BMI	NAFLD	Median 6.4 years	CV events; all-cause mortality
Lan (2022) [39]	China	Prospective cohort (Kailuan)	73,907	<23 kg/m²	NAFLD	Median 13.1 years	Incident CVD; all-cause mortality
Choi (2023) [40]	South Korea	Hospital-based cohort	1,363	Lean/normal weight	MAFLD	Median 39.1 months	ASCVD; advanced fibrosis
Chung (2023) [41]	South Korea	Population-based retrospective (NHIS)	9,935,314	<23 kg/m²	MAFLD	Median 9.3 years	All-cause, CV, cancer, liver mortality
Ishido (2023) [18]	Japan	Single-center retrospective	581	<23 kg/m²	NAFLD	3 years	Incident CVD
Wijarnpreecha (2023) [10]	USA	Single-center retrospective (U. Michigan, 2012-2021)	13,420	Ethnicity-specific	NAFLD	Median 49.3 months	Mortality; incident CVD
Xu (2023) [42]	China	Retrospective cohort	72,392	<23 kg/m²	MAFLD	Median 5.22 years	CHD; stroke; heart failure
Dallio (2025) [43]	Italy	Retrospective longitudinal (single center, 2016-2021)	931 (lean 134; non-lean 797)	<25 kg/m²	MASLD/MAFLD	3 years	Acute CV events
Ghani (2025) [8]	USA	Multicenter retrospective (Banner + U. Michigan, 2012-2023)	75,921	<25; <23 (Asian)	MASLD	Not reported	Mortality; CVD; MACE
Njei (2025) [7]	USA	Retrospective cohort (VA, 2008-2021)	15,155	<25; <23 (Asian)	MASLD cirrhosis	Median 3.6 years	MACE; CV and all-cause mortality
Zhang, NHANES (2026) [11]	USA	Population-based cohort	14,740	<25 kg/m²	MASLD	Median 7.3 years	All-cause and cause-specific mortality
Zhang, UK Biobank (2025) [44]	UK	Prospective cohort	434,417	<23 kg/m²	MAFLD	Median 12.6 years	Multi-system morbidity; all-cause mortality
Al Ta’ani (2026) [12]	Multinational (TriNetX)	Retrospective, 1:1 propensity-matched	Matched 135,038 (67,519 per group)	<25 kg/m²	MASLD	7 years	CV/cerebrovascular events; heart failure; all-cause mortality

Cohort Overlap

Two potential overlaps were assessed against data source, institution, recruitment period, and eligibility criteria rather than shared authorship.

Golabi et al. [38] analyzed the Third National Health and Nutrition Examination Survey (NHANES III), recruited between 1988 and 1994, whereas Zhang et al. [11] analyzed contemporary NHANES cycles. The two studies draw on different survey cycles and different participants, and they are therefore not treated as overlapping.

Wijarnpreecha et al. [10] analyzed a University of Michigan cohort recruited between 2012 and 2021, and Ghani et al. [8] analyzed a combined Banner Health and University of Michigan cohort recruited between 2012 and 2023. These populations genuinely overlap in institution and recruitment period. Wijarnpreecha et al. contributed to the all-cause mortality and composite cardiovascular analyses; Ghani et al. contributed to neither, being ineligible for pooling on separate methodological grounds. No participant is therefore counted twice within any single pooled analysis.

Risk of Bias

**Figure 2:**
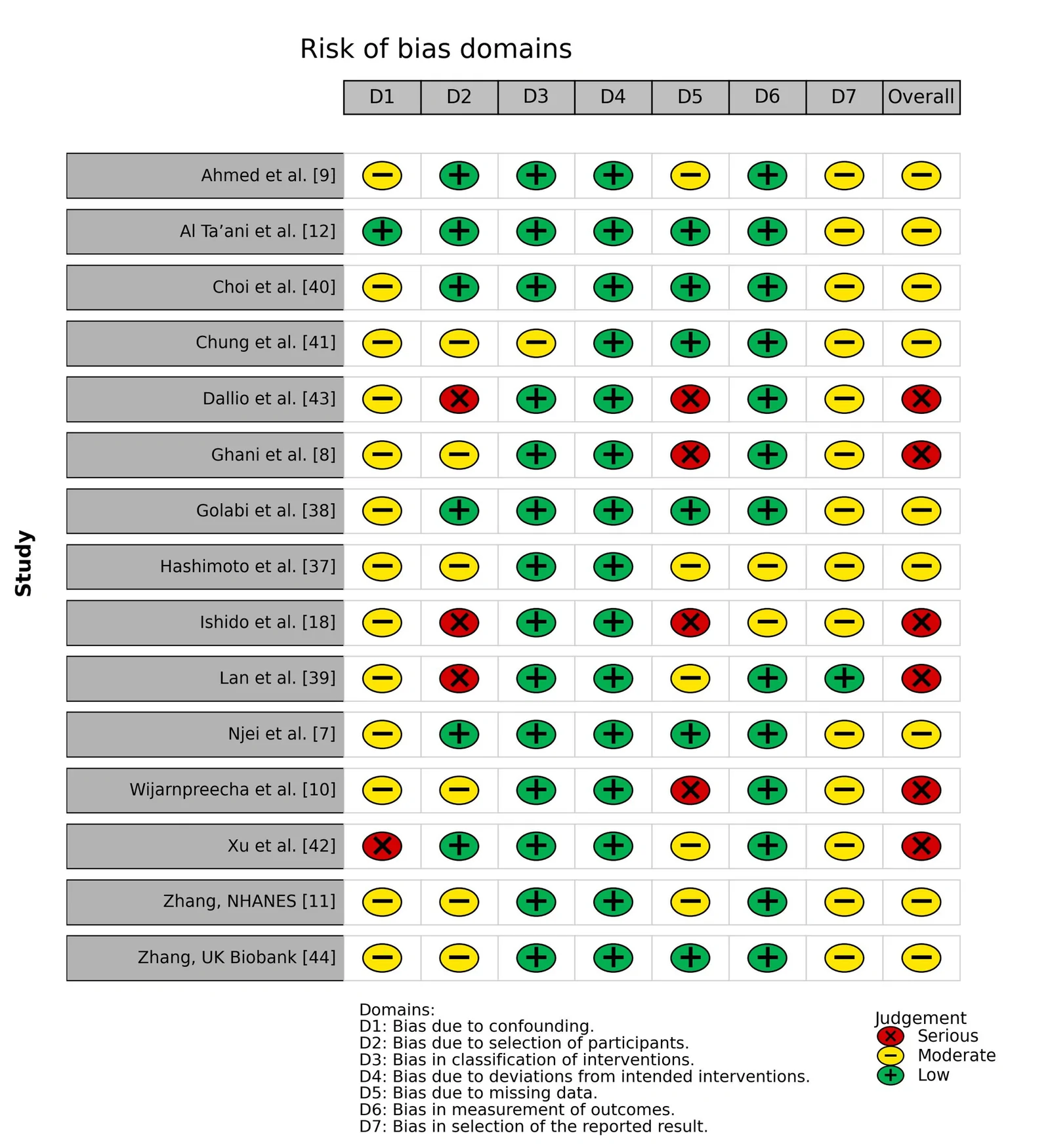
Risk-of-bias traffic-light plot for included studies. Study-level ROBINS-I judgments across the seven domains and overall risk of bias for the 15 included studies: Ahmed et al. [9], Al Ta’ani et al. [12], Choi et al. [40], Chung et al. [41], Dallio et al. [43], Ghani et al. [8], Golabi et al. [38], Hashimoto et al. [37], Ishido et al. [18], Lan et al. [39], Njei et al. [7], Wijarnpreecha et al. [10], Xu et al. [42], Zhang, NHANES [11], and Zhang, UK Biobank [44]. Green indicates low risk, yellow indicates moderate risk, and red indicates serious risk. Domains use exposure-specific adaptation: D3 refers to the classification of lean versus non-lean exposure, and D4 refers to differential management or deviations after exposure classification. ROBINS-I: Risk Of Bias In Non-randomized Studies of Interventions

No study was judged to be at low overall risk of bias. Ten studies were rated at moderate overall risk and five at serious overall risk. Study-level judgments are presented in Table 3 and Figure 2. Most studies had important methodological concerns, particularly residual confounding, participant selection, missing data, and selective reporting.

**Table 3:** Risk-of-bias judgments by study and domain, using ROBINS-I with exposure-specific domain adaptation. ROBINS-I: Risk Of Bias In Non-randomized Studies of Interventions

Study	D1	D2	D3	D4	D5	D6	D7	Overall
Ahmed (2022) [9]	Moderate	Low	Low	Low	Moderate	Low	Moderate	Moderate
Al Ta’ani (2026) [12]	Low	Low	Low	Low	Low	Low	Moderate	Moderate
Choi (2023) [40]	Moderate	Low	Low	Low	Low	Low	Moderate	Moderate
Chung (2023) [41]	Moderate	Moderate	Moderate	Low	Low	Low	Moderate	Moderate
Dallio (2025) [43]	Moderate	Serious	Low	Low	Serious	Low	Moderate	Serious
Ghani (2025) [8]	Moderate	Moderate	Low	Low	Serious	Low	Moderate	Serious
Golabi (2020) [38]	Moderate	Low	Low	Low	Low	Low	Moderate	Moderate
Hashimoto (2017) [37]	Moderate	Moderate	Low	Low	Moderate	Moderate	Moderate	Moderate
Ishido (2023) [18]	Moderate	Serious	Low	Low	Serious	Moderate	Moderate	Serious
Lan (2022) [39]	Moderate	Serious	Low	Low	Moderate	Low	Low	Serious
Njei (2025) [7]	Moderate	Low	Low	Low	Low	Low	Moderate	Moderate
Wijarnpreecha (2023) [10]	Moderate	Moderate	Low	Low	Serious	Low	Moderate	Serious
Xu (2023) [42]	Serious	Low	Low	Low	Moderate	Low	Moderate	Serious
Zhang, NHANES (2026) [11]	Moderate	Moderate	Low	Low	Moderate	Low	Moderate	Moderate
Zhang, UK Biobank (2025) [44]	Moderate	Moderate	Low	Low	Low	Low	Moderate	Moderate

**Figure 3:**
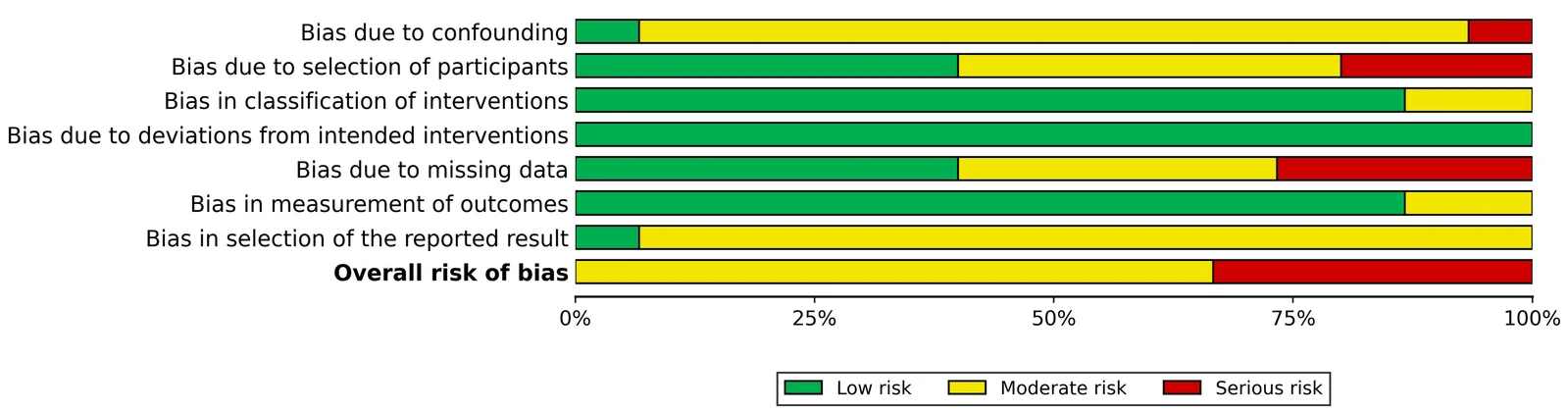
Risk-of-bias summary plot. Proportion of the 15 included studies classified as low, moderate, or serious risk of bias across each domain and overall. Proportions are unweighted counts of studies. Domain labels use the exposure-specific adaptation described in the Methods.

Concerns were concentrated in two domains. Bias due to confounding (D1) was rated moderate in 13 studies and serious in one, and bias in selection of the reported result (D7) was rated moderate in 14 of 15 studies. Serious ratings arose predominantly from bias in selection of participants (D2, three studies) and bias due to missing data (D5, four studies). Classification of the exposure (D3) and measurement of outcomes (D6) were rated at low risk in most studies, while deviations after exposure classification (D4) were rated at low risk in all 15 studies. The concentration of concern in the confounding domain is expected in this literature: leanness is not randomized, and the metabolic characteristics distinguishing lean from non-lean patients are also determinants of cardiovascular outcome (Table 4 and Figure 3).

**Table 4:** Distribution of risk-of-bias judgments by domain.

Domain	Low	Moderate	Serious
D1: Bias due to confounding	1	13	1
D2: Bias in selection of participants	6	6	3
D3: Bias in classification of the exposure	13	2	0
D4: Bias due to deviations after exposure classification	15	0	0
D5: Bias due to missing data	6	5	4
D6: Bias in measurement of outcomes	13	2	0
D7: Bias in selection of the reported result	1	14	0
Overall	0	10	5

All-Cause Mortality

**Figure 4:**
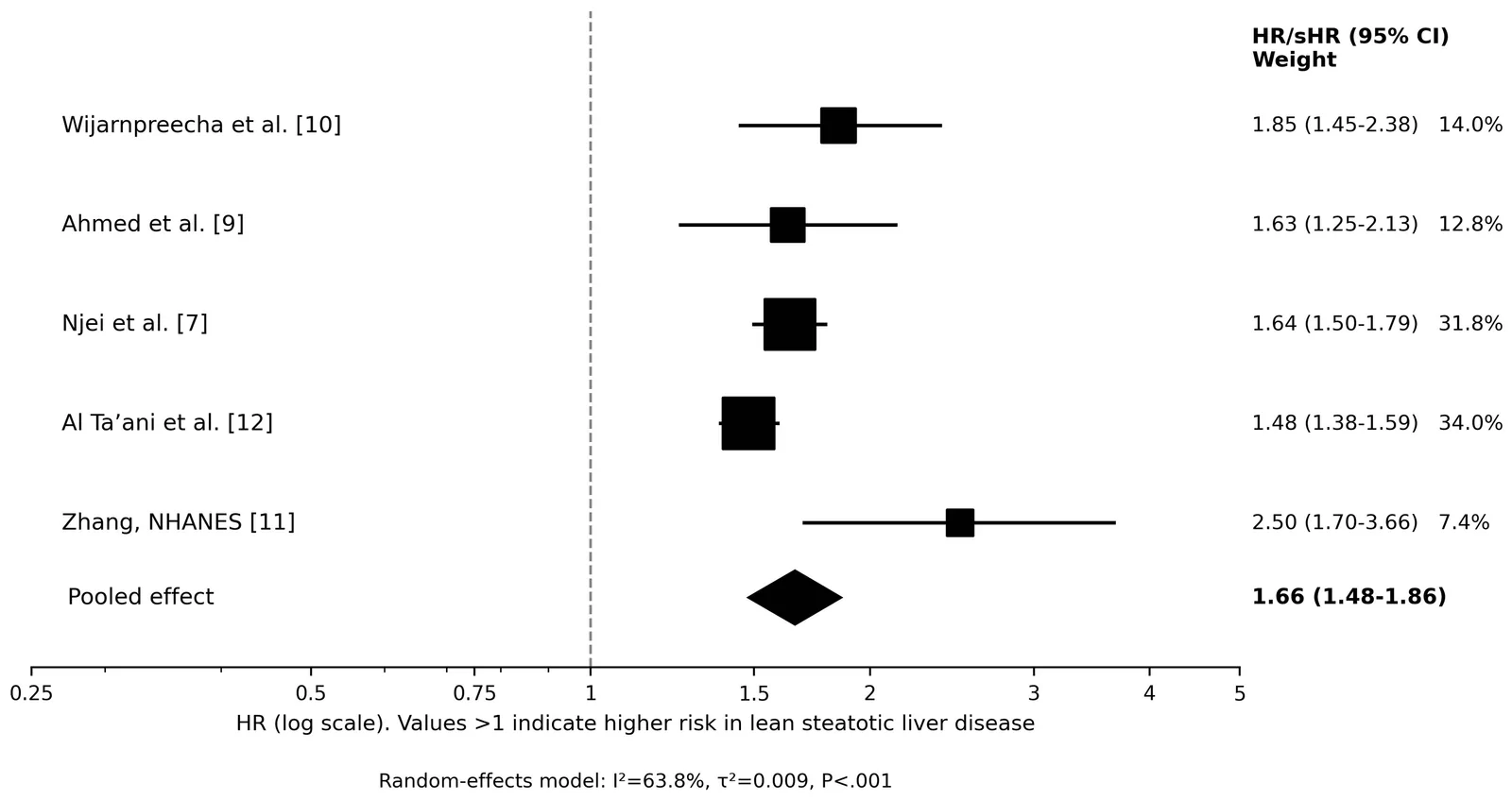
Forest plot for all-cause mortality. Pooled hazard ratio for all-cause mortality comparing lean versus non-lean or higher-body-mass-index steatotic liver disease. Hazard ratios (HRs) are presented on a logarithmic scale; values above 1 indicate higher mortality in the lean group. A random-effects DerSimonian-Laird model was used. The pooled estimate was 1.66 (95% CI, 1.48-1.86); P<0.001 for overall effect; I²=63.8%; τ²=0.009; heterogeneity P=0.03; five contributing studies: Wijarnpreecha et al. [10], Ahmed et al. [9], Njei et al. [7], Al Ta’ani et al. [12], and Zhang, NHANES [11]. NHANES: National Health and Nutrition Examination Survey; sHR: subdistribution hazard ratio

Five studies contributed adjusted HRs for all-cause mortality. The harmonized effect estimates entered into all quantitative analyses are presented in Table 5. Lean steatotic liver disease was associated with higher all-cause mortality than non-lean or higher-body-mass-index comparator groups (pooled HR 1.66; 95% CI, 1.48-1.86; P<0.001; Q=11.05, df=4; heterogeneity P=0.03; I²=63.8%; τ²=0.009). The direction of effect was consistent across all five contributing studies (Figure 4).

**Table 5:** Effect estimates entered into the quantitative analyses. HR: hazard ratio; sHR: subdistribution hazard ratio; CV: cardiovascular; MACE: major adverse cardiovascular events; BMI: body mass index

Study (year)	Analysis	Measure	Original direction	Inverted	Harmonized estimate (95% CI)
Lan (2022) [39]	Composite CV	HR	Overweight/obese vs lean	Yes	0.77 (0.63-0.94)
Wijarnpreecha (2023) [10]	Composite CV	HR	Overweight vs lean	Yes	0.71 (0.51-1.00)
Ahmed (2022) [9]	Composite CV	HR	Lean/normal BMI vs obese	No	1.23 (0.85-1.78)
Njei (2025) [7]	Composite CV (MACE)	sHR	Lean vs non-lean	No	0.79 (0.68-0.93)
Al Ta’ani (2026) [12]	Composite CV	HR	Lean vs non-lean	No	1.21 (1.13-1.30)
Wijarnpreecha (2023) [10]	All-cause mortality	HR	Overweight vs lean	Yes	1.85 (1.45-2.38)
Ahmed (2022) [9]	All-cause mortality	HR	Lean/normal BMI vs obese	No	1.63 (1.25-2.13)
Njei (2025) [7]	All-cause mortality	HR	Lean vs non-lean	No	1.64 (1.50-1.79)
Al Ta’ani (2026) [12]	All-cause mortality	HR	Lean vs non-lean	No	1.48 (1.38-1.59)
Zhang, NHANES (2026) [11]	All-cause mortality	HR	Lean vs non-lean	No	2.50 (1.70-3.66)
Njei (2025) [7]	CV mortality	HR	Lean vs non-lean	No	1.40 (1.12-1.76)
Zhang, NHANES (2026) [11]	CV mortality	sHR	Lean vs non-lean	No	0.94 (0.47-1.89)

Composite Cardiovascular Outcomes and MACE

Five studies contributed estimates for composite cardiovascular outcomes and MACE. Endpoint definitions differed materially between studies and were not all MACE as conventionally defined; components are set out in Table 6. Other contributors to heterogeneity included different body mass index thresholds for leanness, indirect versus direct comparator groups, population source, baseline cardiometabolic risk, adjustment strategy, and the inclusion of one competing-risks sHR alongside conventional Cox HRs. The pooled estimate showed no statistically significant difference between lean and non-lean groups (pooled HR/sHR 0.92; 95% CI, 0.70-1.19; P=0.52; Q=42.8, df=4; heterogeneity P<0.001; I²=90.7%; τ²=0.077).

**Table 6:** Composite cardiovascular endpoint definitions among studies included in the pooled analysis of composite cardiovascular outcomes. MACE: major adverse cardiovascular events

Study	Endpoint as defined in the source report
Lan (2022) [39]	Incident cardiovascular disease (composite)
Wijarnpreecha (2023) [10]	Incident cardiovascular disease
Ahmed (2022) [9]	Cardiovascular events
Njei (2025) [7]	MACE
Al Ta’ani (2026) [12]	Cardiovascular and cerebrovascular events (composite)

Restricting the composite cardiovascular analysis to the four conventional Cox hazard-ratio estimates, excluding the sHR reported by Njei et al. [7], produced a similarly null estimate (HR 0.96; 95% CI, 0.70-1.30; P=0.77; Q=25.29, df=3; heterogeneity P<0.001; I²=88.1%; τ²=0.080).

**Figure 5:**
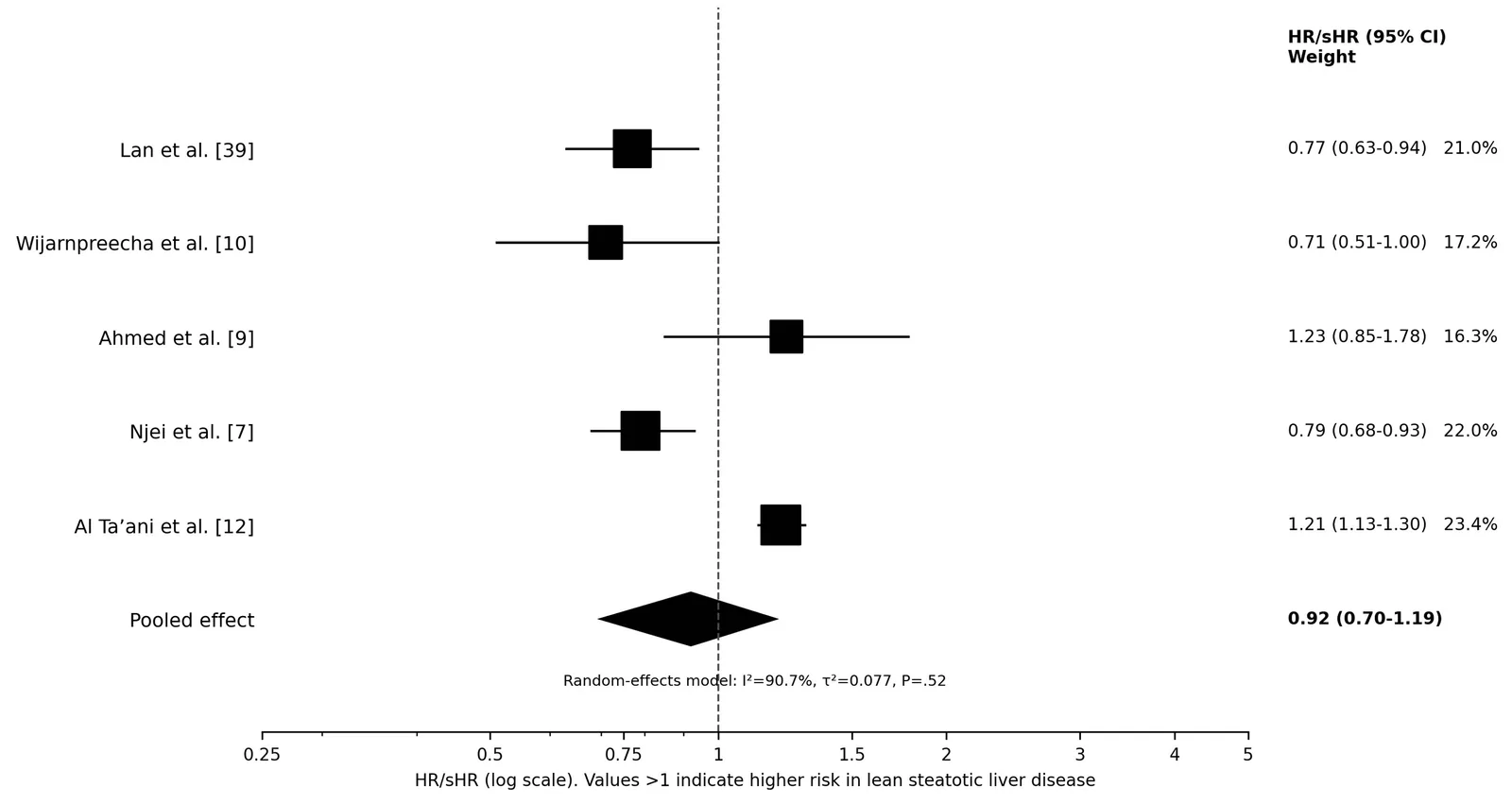
Forest plot for composite cardiovascular outcomes and MACE. Pooled estimate for composite cardiovascular outcomes comparing lean versus non-lean steatotic liver disease. Hazard ratios (HRs) and subdistribution hazard ratios (sHRs) are presented on a logarithmic scale; values above 1 indicate higher risk in the lean group. Estimate type is indicated for each study. A random-effects DerSimonian-Laird model was used. The pooled estimate was 0.92 (95% CI, 0.70-1.19); P=0.52 for overall effect; I²=90.7%; τ²=0.077; heterogeneity P<0.001; five contributing studies: Lan et al. [39], Wijarnpreecha et al. [10], Ahmed et al. [9], Njei et al. [7], and Al Ta’ani et al. [12]. MACE: major adverse cardiovascular events

At this magnitude of heterogeneity, the pooled point estimate conveys limited information: individual study estimates ranged from below to above unity, and between-study variance dominates the model. The composite estimate should therefore be interpreted with considerable caution. No statistically significant difference was observed; however, heterogeneity was substantial, and the estimate is inconclusive rather than evidence of equivalent risk. It should not be interpreted as indicating that the lean phenotype is protective (Figure 5).

Cardiovascular Mortality

**Figure 6:**
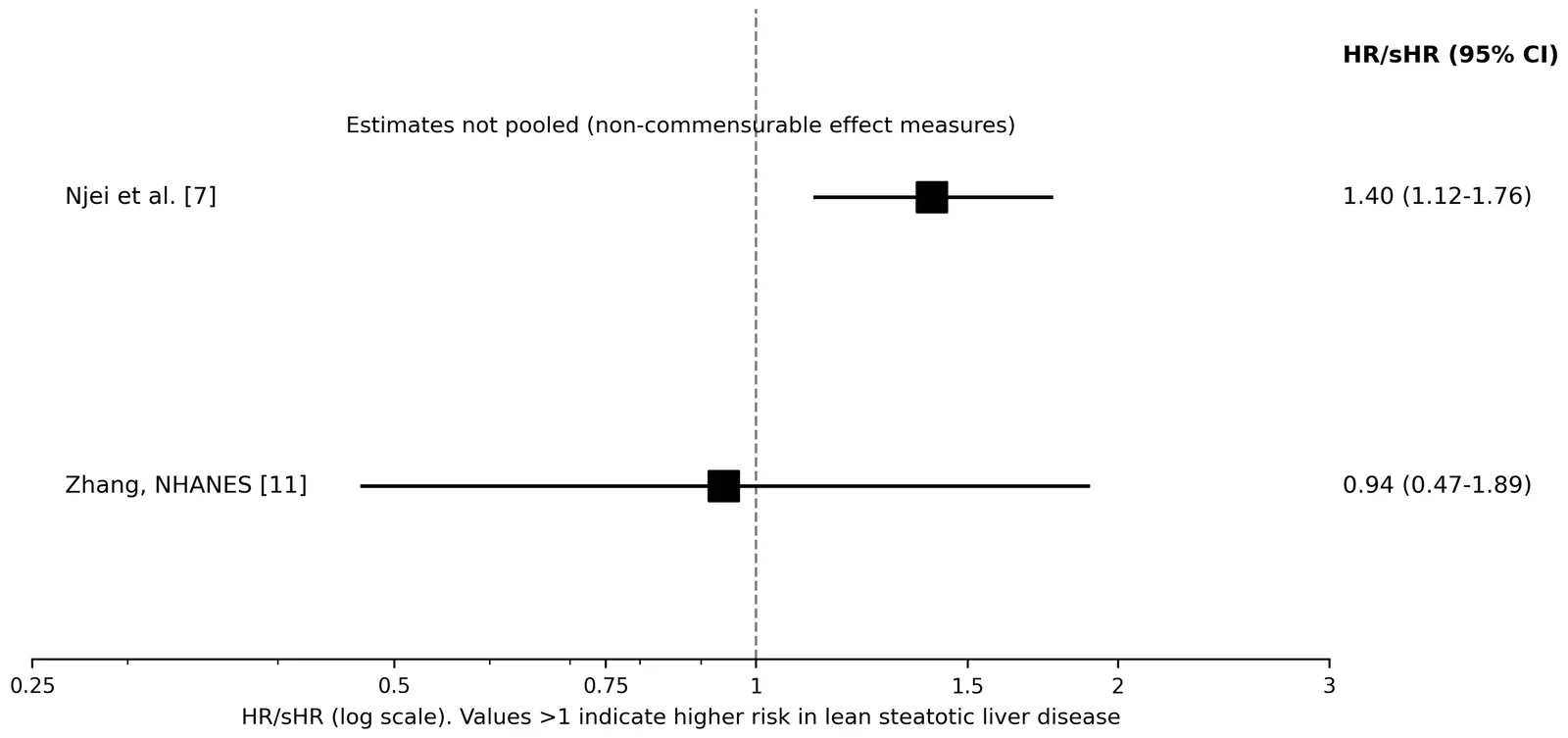
Cardiovascular mortality estimates. Study-level estimates for cardiovascular mortality in lean versus non-lean steatotic liver disease are shown without a pooled diamond because the two available estimates used different effect measures and were not pooled. Njei et al. [7] reported an HR, whereas Zhang, NHANES [11] reported an sHR. Estimates are shown on a logarithmic scale; values above 1 indicate higher risk in the lean group. HR: hazard ratio; sHR: subdistribution hazard ratio; National Health and Nutrition Examination Survey

Cardiovascular mortality was reported by two studies. Njei et al. [7] reported a Cox HR of 1.40 (95% CI, 1.12-1.76), whereas Zhang et al. [11] reported an sHR of 0.94 (95% CI, 0.47-1.89) from a competing-risks model. Because these effect measures represent different estimands in the presence of competing risks, and because only two studies were available, they were not pooled. The two estimates are presented separately in Figure 6. The available evidence is insufficient to determine the direction or magnitude of cardiovascular mortality risk in lean steatotic liver disease. A summary of all quantitative syntheses is presented in Table 7.

**Table 7:** Summary of pooled meta-analysis results. Primary random-effects inverse-variance model with the DerSimonian-Laird estimator. Cardiovascular mortality does not appear in this table because the two available estimates were not pooled. Hartung-Knapp-Sidik-Jonkman sensitivity intervals are reported in the text. k: number of contributing studies; HR: hazard ratio; sHR: subdistribution hazard ratio; CV: cardiovascular; MACE: major adverse cardiovascular events

Outcome	k	Measure	Pooled estimate	95% CI	P	Q (df)	Het. P	I² (%)	τ²
All-cause mortality	5	HR	1.66	1.48-1.86	<0.001	11.05 (4)	0.03	63.8	0.009
All-cause mortality, direct-comparison sensitivity	3	HR	1.64	1.42-1.90	<0.001	9.16 (2)	0.01	78.2	0.011
Composite CV outcomes/MACE	5	HR/sHR	0.92	0.70-1.19	0.52	42.8 (4)	<0.001	90.7	0.077
Composite CV outcomes, HR-only sensitivity	4	HR	0.96	0.70-1.30	0.77	25.29 (3)	<0.001	88.1	0.080

Sensitivity Analyses

Restricting the all-cause mortality analysis to the three studies providing a direct lean-versus-non-lean comparison - Njei et al. [7], Al Ta’ani et al. [12], and Zhang, NHANES (2026) [11] - produced a pooled HR of 1.64 (95% CI, 1.42-1.90; P<0.001; Q=9.16, df=2; heterogeneity P=0.01; I²=78.2%; τ²=0.011). The two studies excluded from this sensitivity analysis were Wijarnpreecha et al. [10], whose estimate was inverted from an overweight-versus-lean contrast, and Ahmed et al. [9], whose comparator was obese rather than a combined non-lean group. The composite cardiovascular HR-only sensitivity analysis is reported above and in Table 7.

Hartung-Knapp-Sidik-Jonkman sensitivity intervals did not materially change the interpretation of the analyses. All-cause mortality remained elevated (HR 1.66; 95% CI, 1.37-2.00; P=0.002), and the direct-comparison sensitivity analysis remained elevated but less precise (HR 1.64; 95% CI, 1.01-2.66; P=0.048). Composite cardiovascular outcomes remained non-significant in both the mixed HR/sHR analysis (HR/sHR 0.92; 95% CI, 0.66-1.27; P=0.50) and the HR-only analysis (HR 0.96; 95% CI, 0.60-1.51; P=0.77).

Narrative Synthesis

Nine included studies were not pooled, for three distinct methodological reasons.

Study whose comparator was fractured across body mass index strata: In Ghani et al. [8], effect estimates were reported separately for overweight, obesity class I, and obesity class II-III, each referenced to the same lean group. Recombining these would have reused the lean reference population multiple times, and no aggregate non-lean estimate was available for inverse-variance weighting.

Studies whose contrast was not lean versus non-lean disease: Hashimoto et al. [37] compared nonoverweight participants with NAFLD against nonoverweight participants without it. Xu et al. [42] set lean MAFLD against participants with no fatty liver. Chung et al. [41] compared MAFLD-lean, MAFLD-overweight/obese, and MAFLD-diabetes subgroups each against a no-MAFLD reference, without comparing the disease groups with one another. Golabi et al. [38] constructed groups from body mass index and waist circumference jointly, producing phenotypes that cannot be mapped onto a lean-versus-non-lean division. Zhang et al. [44] modeled MAFLD subtype jointly with air-pollution exposure, so no isolated lean-versus-non-lean estimate could be recovered. Dallio et al. [43] compared MAFLD with MASLD case definitions within each body mass index stratum rather than comparing lean with non-lean patients.

Studies reporting incompatible effect measures: Ishido et al. [18] reported an adjusted OR, and Choi et al. [40] reported logistic-regression ORs from a hospital cohort; neither can be placed on the same scale as an adjusted HR. The studies retained for narrative synthesis, and the reasons they were not quantitatively pooled, are summarized in Table 8.

**Table 8:** Included studies retained for narrative synthesis, grouped by reason for non-pooling.

Group	Studies	Reason not pooled
Comparator fractured across BMI strata	Ghani (2025) [8]	Estimates split across BMI classes sharing one lean reference; no aggregate non-lean estimate
Indirect comparator	Hashimoto (2017) [37]; Golabi (2020) [38]; Chung (2023) [41]; Xu (2023) [42]; Dallio (2025) [43]; Zhang, UK Biobank (2025) [44]	Contrast is against a non-disease group, a jointly defined phenotype, or a different case definition rather than non-lean disease
Incompatible effect measure	Ishido (2023) [18]; Choi (2023) [40]	Adjusted odds ratios, not commensurable with time-to-event hazard ratios

The narrative evidence was broadly directionally consistent for mortality: Chung et al. [41] reported higher all-cause mortality in the MAFLD-lean subgroup (HR 1.36; 95% CI, 1.34-1.38) than in the MAFLD-overweight/obese subgroup (HR 1.19; 95% CI, 1.18-1.20), each relative to a no-MAFLD reference, a pattern consistent in direction with the pooled mortality estimate although not a direct lean-versus-non-lean contrast. Cardiovascular findings remained variable across these studies, with no consistent direction.

Small-Study Effects

Formal testing was not conducted. No outcome reached the threshold of 10 contributing studies below which funnel-plot asymmetry and regression-based tests are unreliable.

Discussion

Three outcomes behaved differently, and that difference is the substantive result of this review. All-cause mortality was consistently and substantially higher among lean patients, with a pooled estimate robust to restriction of the analytic set, although the precision of this conclusion was reduced under small-sample-adjusted random-effects inference. Composite cardiovascular events showed no significant difference, but with heterogeneity so extreme that the pooled figure carries little interpretive weight. Cardiovascular mortality could not be synthesized at all, because the only two available estimates used non-commensurable effect measures. The cardiovascular mortality evidence should therefore be regarded as insufficient rather than neutral. A review that had merged these three outcomes into a single cardiovascular composite would have reported a null or near-null result and concealed the mortality signal entirely.

The divergence between elevated all-cause mortality and unchanged cardiovascular event rates admits at least two readings. Either lean patients die more frequently of causes other than incident cardiovascular events, or cardiovascular events are differentially ascertained or survived across phenotypes. The present data cannot distinguish between these possibilities.

The pattern reported here is broadly congruent with earlier syntheses, although the magnitude of the mortality estimate is larger than in most. A meta-analysis of lean versus non-lean NAFLD encompassing more than one million individuals found similar adjusted cardiovascular risk between phenotypes (HR 0.89; 95% CI, 0.77-1.02) alongside worse liver-related outcomes, and similar cardiovascular mortality (HR 1.26; 95% CI, 0.89-1.78) [13]. That cardiovascular null closely resembles our own. A separate synthesis restricted to mortality reported a pooled HR of 1.34 (95% CI, 1.23-1.47) for all-cause mortality among lean patients [14], directionally identical to our estimate of 1.66 but smaller in magnitude. Our higher estimate probably reflects the inclusion of two recent large studies with strong effects and a smaller contributing set.

Prior reviews have differed in whether incompatible effect measures were combined, and this decision materially affects the apparent cardiovascular mortality signal. Our decision not to pool an HR with an sHR follows from the same reasoning.

Differences across reviews trace to identifiable sources: the body mass index threshold applied and whether Asian-specific cutoffs were used; whether the reference group is non-lean disease, obesity, overweight, or no disease; baseline diabetes and fibrosis burden; the composition of the cardiovascular composite endpoint; adjustment for cardiometabolic and fibrosis covariates; and whether competing risks were modeled [7,8]. These factors determine whether a lean-versus-non-lean comparison is answering the same question across studies, and they probably contribute to the substantial heterogeneity observed in the composite cardiovascular analysis.

The mechanisms discussed here are hypotheses consistent with the observed associations, not findings of this meta-analysis. Visceral and ectopic adiposity in the presence of a normal body mass index would reconcile normal weight with adverse metabolic risk, and is well documented in lean steatotic liver disease [6]. Sarcopenia and low skeletal muscle mass, more frequent among lean patients, are associated with frailty and mortality and would plausibly contribute to non-cardiovascular death; Choi et al. [40] reported lower muscle mass and handgrip strength in lean patients than in overweight or obese patients with MAFLD, together with a higher estimated atherosclerotic cardiovascular disease risk.

Elevated all-cause mortality without a corresponding excess of cardiovascular events may reflect competing causes of death, including malignancy, liver-related death, frailty, and smoking-related disease. Chung et al. [41] reported markedly elevated liver-related mortality in the MAFLD-lean subgroup (HR 2.84; 95% CI, 2.72-2.97), which is consistent with this reading. Residual confounding by unmeasured factors, notably smoking and occult alcohol consumption, could also inflate the mortality estimate, since neither was consistently adjusted for across the contributing cohorts.

A normal body mass index does not indicate a benign prognosis in steatotic liver disease, and body mass index alone is inadequate for risk stratification in this population. This evidence does not support any specific screening interval, diagnostic pathway, or therapeutic intervention, none of which it can adjudicate. It does suggest that lean patients should not be triaged to lower-intensity metabolic or hepatic assessment on the basis of weight.

The review has a prospectively registered protocol, a systematic search across three databases, duplicate screening and extraction, and formal risk-of-bias appraisal. Its principal methodological strengths lie in what it declined to do: outcomes were analyzed separately rather than merged; incompatible effect measures were kept out of pooled models rather than combined to inflate study counts; effect directions were harmonized explicitly; and studies sharing a lean reference group across body mass index strata were not mathematically recombined.

All contributing evidence is observational, and residual confounding cannot be excluded even where adjusted estimates were used. No study was at low overall risk of bias. Definitions of leanness, of the non-lean comparator, and of the cardiovascular composite varied across studies, and the included cohorts mix MASLD, MAFLD, and NAFLD criteria without formal reclassification, so the findings apply to metabolically defined steatotic liver disease broadly rather than to MASLD as strictly defined. This definitional heterogeneity may influence pooled estimates by combining cohorts with different metabolic entry criteria, different body mass index thresholds, and different comparator structures. Subgroup analyses by disease definition or body mass index threshold were not performed because too few studies contributed to each outcome and because classification features were not independent across studies. Heterogeneity for the composite cardiovascular outcome was extreme, and that pooled estimate should not be relied upon. Only six studies contributed to any pooled analysis, and cardiovascular mortality could not be synthesized. With three to five studies per analysis, conventional random-effects CIs are likely too narrow, although Hartung-Knapp-Sidik-Jonkman sensitivity intervals did not change the qualitative interpretation. Fifteen reports could not be retrieved and were never assessed, a source of potential selection bias that cannot be quantified. Publication bias could not be assessed. Two contributing cohorts draw on partially overlapping institutional populations, although no participant contributes twice to any single pooled analysis. Individual-participant data were unavailable, and no subgroup analysis or meta-regression was adequately powered.

What is needed is prospective cohorts applying contemporary MASLD criteria with region-appropriate body mass index thresholds, direct body-composition measurement, staged fibrosis assessment, adjudicated and consistently defined cardiovascular endpoints, competing-risks analysis for cause-specific mortality, and transparent reporting of confounder adjustment. Reporting cause-specific mortality alongside all-cause mortality would directly address the divergence this review identifies.

## Conclusions

In this synthesis of 15 observational cohort studies, lean steatotic liver disease was associated with higher all-cause mortality than the non-lean phenotype, and this association was robust to restriction to direct comparisons, although precision decreased under small-sample-adjusted inference. Composite cardiovascular event risk did not differ significantly between phenotypes, but heterogeneity was substantial, and the estimate is inconclusive rather than evidence of equivalence. Evidence for cardiovascular mortality was insufficient, being limited to two studies reporting incompatible effect measures. These are associations and not causal effects: all contributing evidence is observational, no study was at low overall risk of bias, and definitions varied across studies. Standardized prospective cohorts using contemporary MASLD criteria, region-appropriate body mass index thresholds, direct body-composition assessment, and adjudicated endpoints are required before the cardiovascular risk of the lean phenotype can be regarded as settled.

## Appendices

Database search strategy

The search was conducted from database inception through July 2026. PubMed, Scopus, and the Cochrane Library were searched, and no restriction on the date of publication was applied.

The search returned 395 records before duplicate removal, including 129 records from PubMed, 253 from Scopus, and 13 from the Cochrane Library. After removing 130 duplicate records, 265 records remained for screening. After removing 130 duplicate records, 265 records were screened based on title and abstract. Forty-eight reports were sought for retrieval; 15 could not be obtained, and 33 reports were assessed for eligibility.

**Table 9:** Database search strategy.

Database	Records retrieved	Complete search string
PubMed	129	(("Non-alcoholic Fatty Liver Disease"[Mesh] OR "fatty liver"[tiab] OR "hepatic steatosis"[tiab] OR NAFLD[tiab] OR MAFLD[tiab] OR MASLD[tiab] OR "metabolic dysfunction-associated fatty liver disease"[tiab] OR "metabolic associated fatty liver disease"[tiab] OR "metabolic dysfunction-associated steatotic liver disease"[tiab] OR "steatotic liver disease"[tiab]) AND (lean[tiab] OR nonobese[tiab] OR "non-obese"[tiab] OR "non obese"[tiab] OR "normal weight"[tiab] OR "normal-weight"[tiab] OR "normal BMI"[tiab] OR "body mass index"[tiab]) AND ("Cardiovascular Diseases"[Mesh] OR cardiovascular[tiab] OR cardiac[tiab] OR coronary[tiab] OR stroke[tiab] OR cerebrovascular[tiab] OR "myocardial infarction"[tiab] OR MACE[tiab] OR mortality[tiab] OR death[tiab] OR survival[tiab]))
Scopus	253	TITLE-ABS-KEY(("fatty liver" OR "hepatic steatosis" OR NAFLD OR MAFLD OR MASLD OR "metabolic dysfunction-associated fatty liver disease" OR "metabolic associated fatty liver disease" OR "metabolic dysfunction-associated steatotic liver disease" OR "steatotic liver disease") AND (lean OR nonobese OR "non-obese" OR "non obese" OR "normal weight" OR "normal-weight" OR "normal BMI" OR "body mass index") AND (cardiovascular OR cardiac OR coronary OR stroke OR cerebrovascular OR "myocardial infarction" OR MACE OR mortality OR death OR survival))
Cochrane Library	13	(("fatty liver" OR "hepatic steatosis" OR NAFLD OR MAFLD OR MASLD OR "metabolic dysfunction-associated fatty liver disease" OR "metabolic associated fatty liver disease" OR "metabolic dysfunction-associated steatotic liver disease" OR "steatotic liver disease") AND (lean OR nonobese OR "non-obese" OR "non obese" OR "normal weight" OR "normal-weight" OR "normal BMI" OR "body mass index") AND (cardiovascular OR cardiac OR coronary OR stroke OR cerebrovascular OR "myocardial infarction" OR MACE OR mortality OR death OR survival))
